# Polysaccharide Nanoparticles from *Isatis indigotica* Fort. Root Decoction: Diversity, Cytotoxicity, and Antiviral Activity

**DOI:** 10.3390/nano12010030

**Published:** 2021-12-23

**Authors:** Guanzhen Gao, Chuanqi He, Huiqin Wang, Jingke Guo, Lijing Ke, Jianwu Zhou, Pik Han Chong, Pingfan Rao

**Affiliations:** 1 Food Nutrition Science Centre, School of Food Science and Biotechnology, Zhejiang Gongshang University, Hangzhou 310012, China; gaoguanzhen@zjgsu.edu.cn (G.G.); huiqinwang@zjgsu.edu.cn (H.W.); lijingke@zjgsu.edu.cn (L.K.); hanleychong@zjgsu.edu.cn (P.H.C.); pingfanrao@zjgsu.edu.cn (P.R.); 2 Institute of Biotechnology, Fuzhou University, Fuzhou 350002, China; chuanqihe@tianshenghaowu.com; 3 Department of Food and Biological Engineering, Zhicheng College, Fuzhou University, Fuzhou 350002, China; kengco@sina.com

**Keywords:** nanoparticles, boiling-induced assembly, *Isatis indigotica* Fort., physicochemical property, antiviral activity

## Abstract

It has been revealed that numerous nanoparticles are formed during the boiling preparation of traditional Chinese medical decoctions and culinary soups. They may possess physiological effects different from those of constituent components and are worth paying attention to but are barely noticed and investigated as of yet. In this study, six groups of nanoparticles, whose size ranged from 57 to 300 nm, were successfully isolated from the decoction of *Isatis indigotica* Fort. root, according to their particle size by the means of size-exclusive chromatography. All of the obtained nanoparticles have a high content of polysaccharides, which distinguishes them from the disclosed BLG protein nanoparticles. They also have high similarities in other compositions, surface charge, and stimuli responses. However, four out of these six nanoparticles (F2, F3, F4, and F5) exhibited significant antiviral activity against influenza virus H1N1, and their antiviral activities and cytotoxicity towards MDCK cells varied with their sizes. It suggested that the antiviral efficacy of BLG decoction could also be from its nanoparticles besides its well-known antiviral phytochemicals. It also implied that the biological effects of these polysaccharide nanoparticles, including cytotoxicity and antiviral activity, may be correlative with the physicochemical properties, especially the particle size.

## 1. Introduction

Boiling extraction (decocting) is the earliest and most popular preparation of Chinese traditional herbal medicine. As a traditional food preparation, boiling can harvest a great amount of micro/nanoscale colloidal particles as a result of food components self-assembly [1,2,3,4], which has been well known in food science. It also produces a great influence on the formation of nanoparticles in herbal decoctions. As reported in previous studies, colloid-like aggregates were observed in all the decoctions of 60 medicinal herbs and 24 Chinese herbal formulae [5], nanoparticles were formed and isolated from the Ma-Xing-Shi-Gan decoction after boiling preparation [6].

To date, although attempts have been made to explore the linkage between nanoparticles and the efficacies of herbal decoctions, it has not been fully revealed yet. Zhang et al. disclosed that the decoction aggregates can pass through the gastrointestinal Caco-2 cell monolayer and exhibited activities correlated to those decoctions [5,7]. A closer inspection on the components of nanoparticles from the Ma-Xing-Shi-Gan decoction showed that the nanoparticles serve as drug vehicles that carry principle bioactive phytochemicals such as ephedrine, pseudoephedrine, and the others [6] to display similar biological function with the decoction itself. Besides, the small size of nanoparticles, namely the nano-size effect, also endows them with unique physicochemical properties and consequent physiology effects [8,9], which are not connected with decoctions’ efficacies or even side effects yet.

Even though these boiling-induced nanomaterials have been consumed from the period of ancient civilizations itself [2], they may possess physiological effects different from those of constituent components and are worth paying attention to but are barely noticed and investigated as of yet. They are the simplest and commonest nanoparticles and this has made it impossible for nanoscientists to associate them with anything of that level of scientific and technical sophistication. Besides, a look at nanoparticles within culinary or medical soup will unfold their instability, complexity in constituent components, and diversity in morphology, which also leads to hardly anyone believing in their maneuverability as fancy nanocarriers.

The sun-dried roots of *Isatis indigotica* Fort., known as *Radix Isatidis* or Ban-Lan-Gen in Chinese, are widely used as a medicinal herb for treating infectious and inflammatory diseases, including influenza, acute hepatitis, herpes, and encephalitis B [10,11]. Its decoction (preparation by boiling) and granules (the dried powder of decoction) are its main type of administration formula in the clinic (China Pharmacopeia Committee, 2015) [12]. Our previous study has revealed that the Ban-Lan-Gen decoction contains a great number of nanoparticles, and their constitutive proteins can assemble into nanoparticles induced upon boiling [13]. In this study, nanoparticles (NPs) were isolated and fractionated from Ban-Lan-Gen boiling water extracts according to their size by the means of size-exclusion chromatography. Six groups of nanoparticles with an average size ranging from 57 nm to 300 nm were obtained and subjected to the investigation of their physicochemical properties, constitutive compounds, cytotoxicities, and antiviral activities.

## 2. Material and Methods

### 2.1. Raw Materials, Chemicals, and Cell Lines

The sun-dried roots (*Radix Isatidis*, Ban-Lan-Gen, BLG) of *Isatis indigotica* Fort. were collected from a GAP (Good Agricultural Practice) field in Fuyang (China). All the chemicals used in this study were reagent/analytical grade from Sinopharm Chemical Reagent Co., Ltd. (Shanghai, China). MTT (Sigma-Aldrich, St. Louis, MO, USA), Madin–Darby Canine Kidney (MDCK) cells were purchased from the Type Culture Collection of the Chinese Academy of Science (Shanghai, China).

### 2.2. BLG Decoction Preparation

The BLG decoction was prepared as described by Zhou et al. [13]. Briefly, sliced roots were soaked in distilled water (1:8, *w*/*v*) for 30 min at room temperature with constant stirring. It was then cooled down to room temperature after boiling for 60 min and the filtrate was obtained through two layers of cotton gauze. The filtrate was centrifuged at 5000 *g* for 10 min and the supernatant was kept for further use.

### 2.3. Separation of Nanoparticles from BLG Decoction

The nanoparticles from the BLG decoction were separated using a size-exclusion gel chromatography with dynamic light scattering (SEC-DLS) as described by Zhou et al. [6] with slight modifications. The equipped Sephacryl S-1000 column (1.0 cm × 100 cm) was equilibrated and eluted with 0.02 M Phosphate buffer (pH 5.0) at a flow rate of 0.35 mL/min. The fraction with strong signals at both light scattering and UV280 nm were collected and pooled for further analysis. 

### 2.4. Characterization of BLG Nanoparticles Fractions

The BLG nanoparticles fractions were analyzed by dynamic light scattering (DLS) on a Zetasizer Nanodevice (Malvern Instruments, Worcestershire, UK) to measure the average hydrodynamic diameter and ζ-potential at 25 °C [14]. The average hydrodynamic diameters of BLG nanoparticles fractions were evaluated by DLS in different pHs (pH = 2, 3, 4, 5, 6, 7, 8, 9, and 10) at 25 °C or different temperatures (20 °C, 30 °C, 40 °C, 50 °C, 60 °C, 70 °C, 80 °C, 90 °C, and 100 °C) using the intensity-weighted mean diameter derived from the cumulants analysis. The conditions of DLS analysis were as follows: dispersant water, dispersion refractive index 1.33, viscosity 0.8872 cP. Before the measurement, the samples were equilibrated at every temperature for 5 min in 10 mm cuvettes with caps. Each measurement was conducted in triplicate.

### 2.5. Scanning Electron Microscopy (SEM)

Scanning electron microscopy (SEM) was performed by a Cold Field Emission S-4800 Scanning Electron Microscope (Hitachi, Tokyo, Japan) operated under an acceleration voltage of 5 kV. The particles were collected with a 0.22 μm cellulose acetate membrane and coated with gold using a sputter coater (E-1010, Hitachi Instruments, Tokyo, Japan) to render them electrically conductive. The images were taken with 20 K magnification and a 7.0 mm scale bar.

### 2.6. Major Composition Analysis of BLG Nanoparticles Fractions

The mass of each BLG nanoparticles fractions was determined using the gravimetric analysis of oven-dried samples. The number of polysaccharides in BLG nanoparticles fractions were measured by the anthrone–sulfuric acid assay using anhydrous glucose as a standard sample [15]. The BLG nanoparticles fractions were hydrolyzed with 6 M hydrochloric acid at 110 °C for 24 h. The hydrolyzed solutions were then dried at 60 °C for deacidification. The total amino acid composition of BLG nanoparticles fractions was determined using a method reported by Yang et al. with slight modifications [16]. It was performed on an L-8800 amino acid analyzer (Hitachi Ltd., Tokyo, Japan) with a cation exchange column (4.6 × 60 mm, Hitachi Ltd.). A mixture of amino acid standards (Sigma, St. Louis, MO, USA) was utilized for quantification.

The epigoitrin contents of the fractions were measured as described by An et al. with slight modifications [17]. Briefly, reverse-phase high-performance liquid chromatography (RP-HPLC) was used to determine the epigoitrin content in the fractions with a Daisogel SP-60-5-ODS-RPS column (5 μm, 4.6 mm × 250 mm, OSAKA SODA Co., Ltd., Osaka, Japan). The mobile phase was made with acetonitrile, water, phosphoric acid, and triethylamine according to the ratio of 8.5:90.72:0.73:0.05. The column was eluted with a flow rate of 0.7 mL/min at 30 °C. The wavelength for detection was set to 245 nm.

### 2.7. Cell Culture and Cytotoxicity Assay (MTT Assay)

Madin–Darby Canine Kidney (MDCK) cells were used to evaluate the influence of BLG nanoparticle fractions on the cellular viability by MTT assay [18]. The cells were seeded into 96-well plates at a density of 5 × 10^4^ cells/well in the MEM (Gibco, Life Technologies Corporation, Grand Island, NY, USA) supplemented with 12% fetal bovine serum (BOVOGEN, Victoria, Australia), 1% streptomycin (10,000 μg/mL) (Gibco, Life Technologies Corporation, Grand Island, NY, USA), 1% GlutaMAX (Gibco, Life Technologies Corporation), 1% MEM Non-Essential Amino Acid Solution (Gibco, Life Technologies Corporation), and 1% Sodium Pyruvate (Gibco, Life Technologies Corporation).

Tested samples were adjusted to the serial concentrations (3.58–0.039 mg/mL), added to the cells in 96-well plates, four duplicates for each, and cultured at 37 °C, 5% CO_2_ for 48 h. The cell viability was calculated with the equation below:

(1) $$\mathrm{Cell}\;\mathrm{Viability}\;\left(\%\right)=\frac{OD_{570-sample}}{OD_{570-control}}\times 100$$

### 2.8. Virus Propagation

The human influenza A/PR/8/34 (H1N1) virus was provided by the Guangzhou Institute of Respiratory Disease. The virus was propagated in embryonated chicken eggs as described previously by Brauer and Chen [19]. Virus titers were measured with the 50% tissue culture infectious dose (TCID_50_) method according to the previous study [20]. The titer of 100 TCID_50_/mL was used for the assessment of antiviral activity.

### 2.9. Antiviral Assay

MDCK cells were seeded into 96-well plates at a density of 5 × 10^4^ cells/well and then cultured for 24 h to 100% confluence. The confluent cells were pre-incubated with 200 μL of H1N1 virus suspensions at the titer of 100 TCID_50_/mL for 2 h at 34 °C. Unattached viruses were removed by washing the well twice with PBS. The cells were then treated with 200 μL of fresh medium supplemented BLG nanoparticles fractions at a concentration of 0.05–0.4 mg/mL at 34 °C. The control cell group (uninfected cells in MEM) and the virus group (virus + MEM) were also conducted in this experiment. After incubation for 72 h, the culture medium was discarded and an MTT assay was carried out. The optical densities (*OD*) of the 96-well plate were measured using an enzyme-labeled instrument at 570 nm. The antiviral activity was calculated using the following formula:

(2) $$\mathrm{Antiviral}\;\mathrm{activity}\;\left(\%\right)=\frac{OD_{sample}-OD_{virus}}{OD_{control}-OD_{virus}}\times 100$$

### 2.10. Statistical Analysis

All experiments were conducted in triplicate and data analyses were performed with GraphPad Prism 5.0 (GraphPad Software, Inc., San Diego, CA, USA). The data are presented as mean ± SD (*n* = 3 or 4) for each measurement. A two-way ANOVA test was used to determine differences among treatment means at the 5% significance level.

## 3. Results

### 3.1. Separation of Nanoparticles from BLG Soups

Size-exclusion chromatography in couple with light scattering has been used to separate and characterize a wide range of nanoparticles [6,21,22]. The nanoparticles in the BLG decoction with high similarities in characteristics and compositions were captured by SEC and separated according to their sizes in this study.

**Figure 1:**
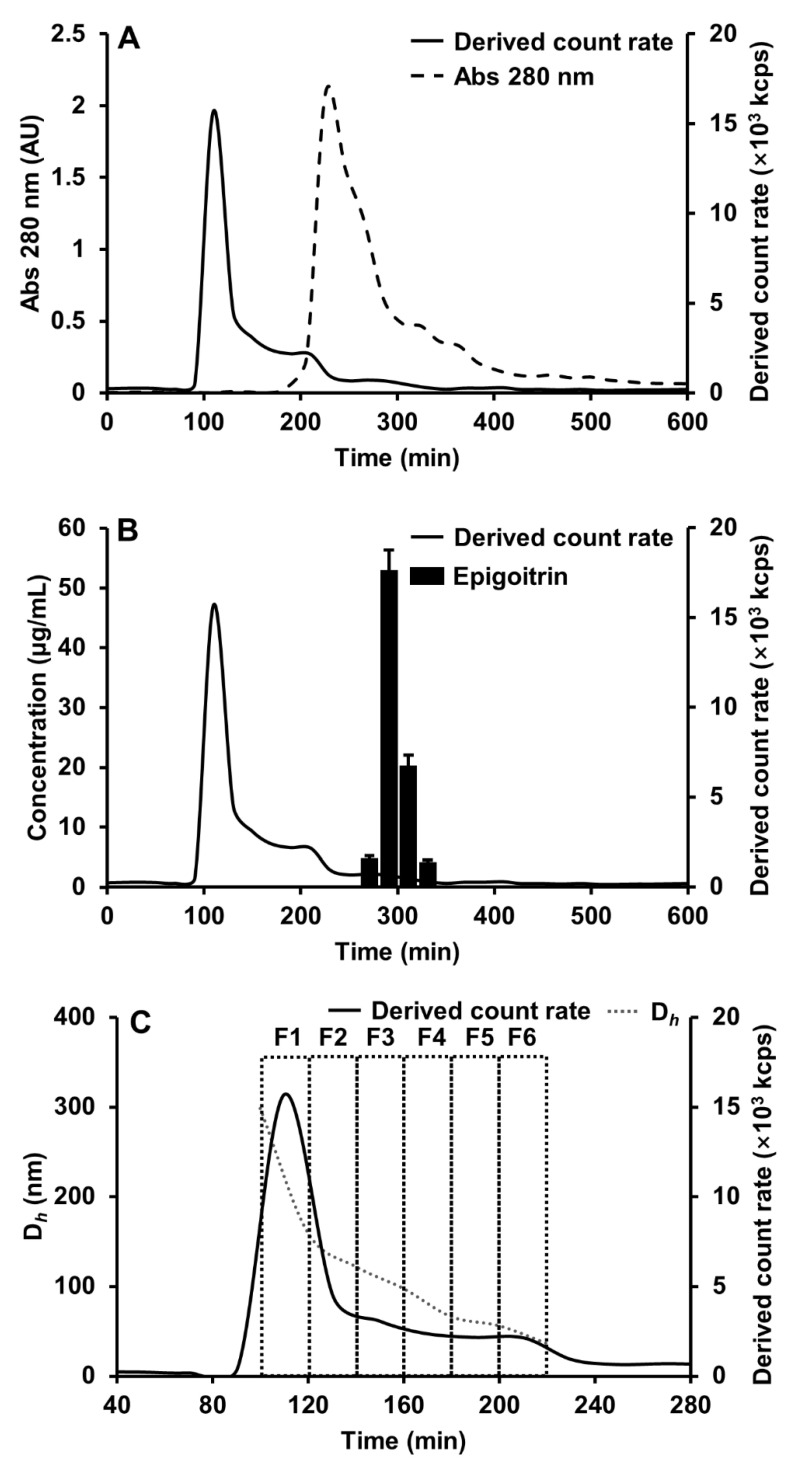
Isolation of BLG nanoparticles fractions derived from the BLG decoction using SEC-DLS. The Sephacryl S-1000 column (1.0 cm × 100 cm) was equilibrated and eluted with 0.02 M Phosphate buffer (pH 5.0) at a flow rate of 0.35 mL/min. (**A**) SEC-DLS isolation of the BLG nanoparticles fractions. (**B**) The content of epigoitrin in the fractions isolated from the BLG decoction. (**C**) The average hydrodynamic diameter radius distributions (dash line) of BLG nanoparticles fractions. Solid line: derived count rate which is light scattering intensity; dashed line: UV absorption at 280 nm; black column: the content of epigoitrin; gray dot line: the average hydrodynamic diameter radius.

As shown in Figure 1, two major peaks with the solid line were observed by light scattering in the chromatogram. The first peak with retention time from 100 to 220 min showed strong light scattering signals but UV absorption at 280 nm was not noticed, indicated by dotted curve. While both light scattering signals and high UV absorption at 280 nm were observed in the second peak with a retention time of 220 min. The strong light scattering intensity indicates the occurrence of particles. Moreover, its overlapping with UV absorption at 280 nm implies the possible participation of protein in the particle assembly. It has been revealed in our previous work that the fraction with a retention time of 220 min, which has both light scattering signal and UV absorption, contains protein-based nanoparticles [13]. However, the first peak that showed no UV absorption was further fractionated into six fractions, denoted as F1 to F6 (see Figure 1C).

### 3.2. Characterization of the Separated BLG Nanoparticle Fractions

As shown in Table 1, the average size of each isolated fraction decreased with the increase of retention time, indicating that SEC is an effective method for separating these nanoparticles according to their particle sizes. The zeta potentials indicated that the nanoparticles within F1–F6 had similar surface charges (see Table 1).

**Figure 2:**
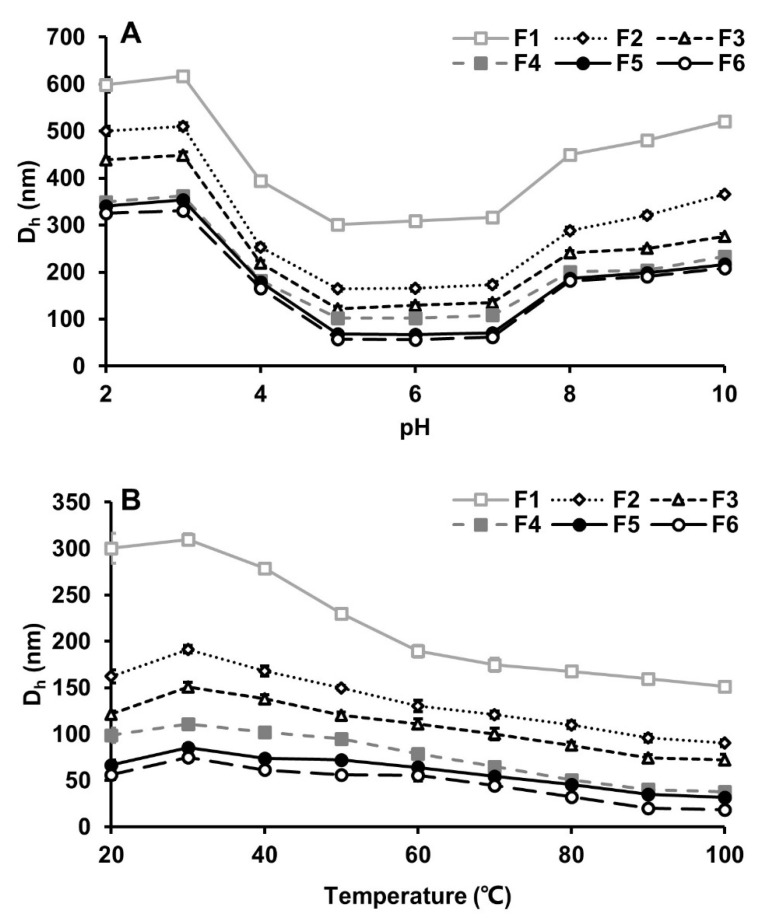
Influences of pH and temperature on BLG nanoparticles derived from BLG decoction. (**A**) The PH response of BLG nanoparticles fractions on the particle D_h_. (**B**) Temperature response of BLG nanoparticles fractions on the particle D_h_.

These nanoparticles were all stimuli sensitive. They exhibited high similarities in temperature and pH response as shown in Figure 2. The average sizes were the largest at 30 °C, either a rise or drop in temperature resulted in decreases of their average sizes, and the smallest average sizes were all observed at 100 °C. The diameter of nanoparticles was decreased with increasing temperature could be explained by the strength of hydrophobic interactions with the temperature fluctuation [23]. At higher temperatures, the hydrophobic interaction becomes stronger, which led to a more compact structure of the nanoparticles’ structure and the diameter becoming smaller [24]. In addition, higher temperatures might cause some compositions to separate from nanoparticles and the diameter to become smaller [25]. Some studies have demonstrated a similar change in β-lactoglobulin/pectin nanoparticle size with increasing temperature [26,27]. Similarly, these nanoparticles exhibited the smallest average sizes at pH 5–7, and the sizes changed to bigger or even doubled sizes at the pH lower than 5 and higher than 7. The hydrodynamic diameter of nanoparticles changed by pH might be attributed to the competition between the Van der Waals and electrostatic interactions among the particles. Lower or higher pH increased the electrostatic repulsion and increased the swelling of the nanoparticles, thereby affecting the hydrodynamic diameter of the nanoparticles. Some studies have demonstrated the mechanism for the influence of pH on nanoparticles’ hydrodynamic diameter [28,29]. Similarities in both surface charge and stimuli response implied that nanoparticles in different fractions may have similar structure and compositions although they are varied in particle size.

A closer inspection disclosed that these isolated nanoparticles have a high content of polysaccharides and small quantities of proteins. As shown in Table 1, total polysaccharides were over 97% of dry matter in all fractions. The proteins were found less than 1% of dry matters in most fractions and it was barely detected in fraction 6. Furthermore, the content of epigoitrin which was considered as a main antiviral component in the Ban-Lan-Gen was screened by reversed-phase chromatography through all fractions from SEC. The results suggested that the nanoparticle fractions, from F1 to F6, do not include epigoitrin, which was only detected from the fractions with retention time ranging from 280 to 340 min, shown in Figure 1B.

**Figure 3:**
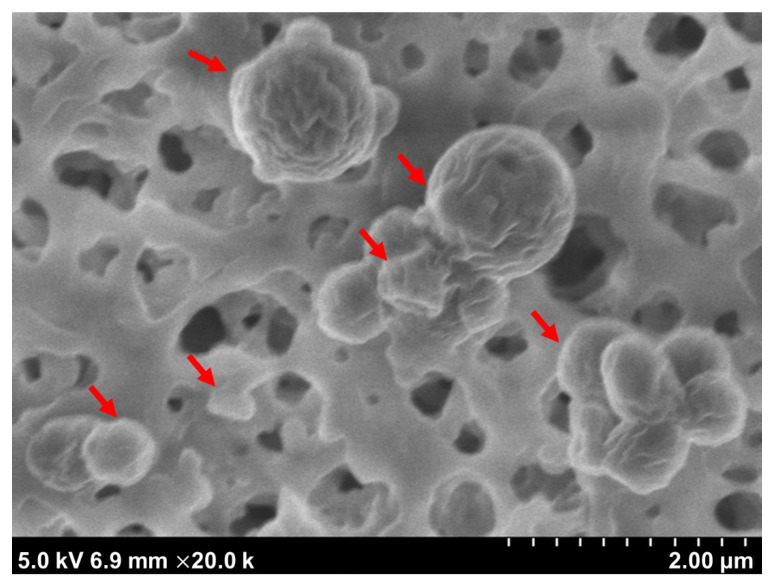
SEM micrograph of BLG nanoparticles within the first peak attached to the surface of a 0.22 μm cellulose acetate membrane. The image shows spherical NPs (indicated with arrows). Magnification ×20,000.

Although their zeta potentials are around −9 mV (shown in Table 1), these nanoparticles exhibited good stability in aqueous suspension. They remained in the same average sizes and light scattering intensities at room temperature (20 °C) after 3 d of storage and formed no visible sediments (data no shown). Moreover, the SEM morphologic observation revealed that the NPs within the first peak are spherical with diameters ranging from a few dozen to a few hundred nanometers (see Figure 3).

### 3.3. Cytotoxicity and Antiviral Activity of BLG Nanoparticle Fractions towards MDCK Cells

**Figure 4:**
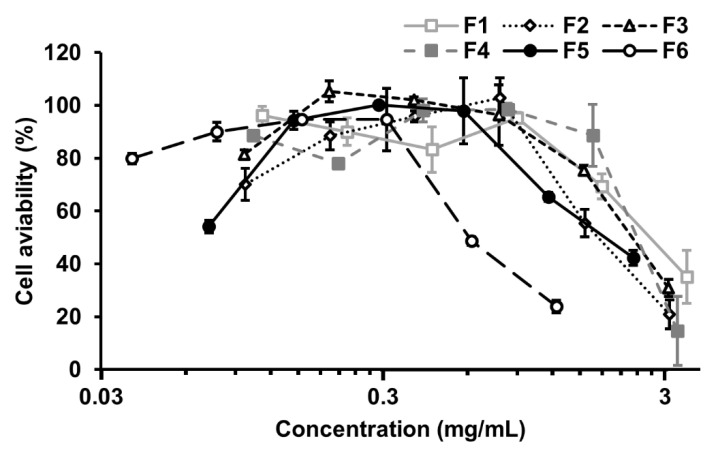
Cytotoxicity of BLG nanoparticles fractions towards MDCK cells. Concentration–response curve to obtain TC_50_ value. Values are expressed as the mean ± SD (*n* = 3).

The toxicities of nanoparticle fractions towards MDCK cells were evaluated by MTT assay, shown in Figure 4 and Table 2. The results indicated that F6 exhibited the smallest TC_50_ (Toxic Concentration 50%) value of 0.67 mg/mL towards MDCK cells, while the biggest value of 2.69 mg/mL was found in F1. The cytotoxicity of nanoparticles is mainly determined by their physicochemical properties [8]. The nanoparticles within F6, which have the smallest average size among the nanoparticle fractions, showed the highest cytotoxicity.

**Figure 5:**
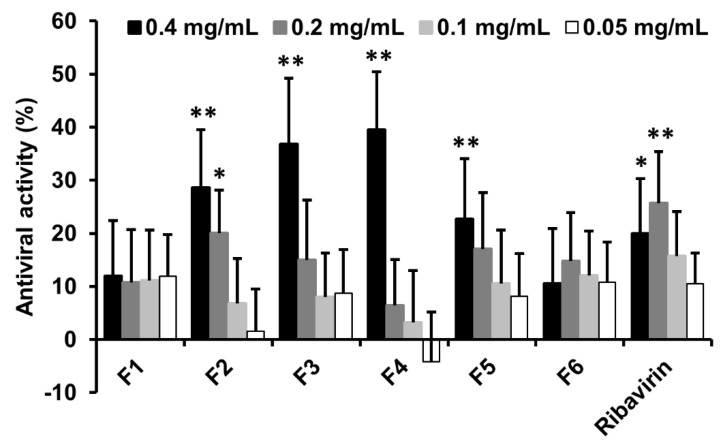
The antiviral effects of BLG nanoparticle fractions against H1N1 infection in the MDCK cells. MDCK cells were pre-incubated with 100 TCID_50_ H1N1 for 2 h at 34 °C and treated with the BLG-NPs fractions at the concentration of 0.05–0.4 mg/mL at 34 °C. MDCK cell viability of cells was evaluated by MTT assay. Values are expressed as the mean ± SD (*n* = 4). * *p* < 0.05, ** p < 0.01 compared to the virus group using the two-way ANOVA followed by Dunnett’s multiple comparisons test.

The antiviral activity of nanoparticle fractions was assessed utilizing a post-treatment assay on infected MDCK cells. As shown in Figure 5, F2, F3, F4, and F5 fractions showed significant antiviral effect at 0.4 mg/mL, even better compared with antiviral agent ribavirin. Among the nanoparticle fractions, the nanoparticles with the largest average size of 300 nm in F1 and the smallest average size of 56 nm in F6 did not exhibit obvious antiviral activity, which implies that the antiviral activity of BLG nanoparticles may be size-dependent within the range from 67 to 162 nm. The antiviral activities of F2, F3, and F4 increased with the decrease of the average size, but the nanoparticles in F5 with smaller sizes instead showed weaker antiviral activity. The results suggested that the antiviral activity of BLG-NPs against influenza virus H1N1 was influenced and determined by their physicochemical properties.

## 4. Discussion

It has been revealed that there are numerous nanoparticles in a variety of traditional Chinese medical decoctions and culinary soups [6,30,31,32]. These nanoparticles, including the BLG nanoparticles in this study, are formed during the boiling preparation, and their formation results from regular food preparation rather than the subjective nanoparticle preparations [33]. The investigations on their constituent components, self-assembly behavior, cytotoxicity, and bioactivity could provide not only a new perspective for the “food–body” interaction [34,35] but also a prototype system for nanomaterials. The studies on the nanoparticles derived from TCM decoctions have navigated the fabrication of protein nanoparticles by using proteins from Radix Glycyrrhizae [3] and *Semen Armeniacae Amarum* [4].

The roots of *Isatis indigotica* fort are rich in amphipathic molecules, including protein [13,36], polysaccharides [37,38], and other active compounds [39]. After being released and migrated into the water during the boiling process, these amphipathic molecules would self-assemble into nanoparticles via non-covalent interactions such as ion bonds, hydrogen bonds, hydrophobic interactions, and Van der Waals forces. In this study, six groups of the BLG nanoparticles that showed strong light scattering signals without UV absorption at 280 nm were isolated by the means of size-exclusive chromatography. The isolated nanoparticles (F1–F6) have a high content of polysaccharides with the total polysaccharide up to 97% of dry matter. Four out of six polysaccharide nanoparticles (F2, F3, F4, and F5) exhibited significant antiviral activity against the influenza virus H1N1. Natural polysaccharides have been widely adopted for the fabrication of nanoparticles due to their advantages on low toxicity, low immunogenicity, good biocompatibility, and so on [40]. The polysaccharide nanoparticles have high efficiency in delivering substances and the potential on targeted delivery due to the specific binding ability to cell surface markers of several types of polysaccharides such as hyaluronic acid and pullulan [41,42]. However, being a naturally occurring nanomaterial, the polysaccharide nanoparticles within BLG decoction may represent a prototype of engineered polysaccharide nanoparticles. An investigation on their physicochemical properties and bioactivities would reveal clues for the fabrication of safer and more efficient nanoparticles, moreover, provide new insight into the antiviral activity of BLG decoctions.

*Isatidis Radix* and its decoction have been employed in clinical practice for the treatment of virus infection and inflammation, e.g., seasonal flu. Its extract exhibited antiviral activity against the influenza virus [43,44]. The polysaccharide nanoparticles also exhibited high antiviral activity against human influenza virus H1N1 in the current study, suggesting that the antiviral efficacy of *Isatidis Radix* decoction would also be attributed to its nanoparticles rather than its well-known phytochemicals. The polysaccharide nanoparticle from BLG decoction is not the sole example, which shows the same bioactivity as the decoction. The nanoparticles in the Bai-Hu-Tang decoction exhibited the same antipyretic effect compared with Bai-Hu-Tang itself [30]. The nanoparticle from freshwater clam (*Corbicula fluminea* Muller) soup performed a hepatoprotective effect in vivo [45]. The nanoparticles from the bone soup can directly interact with macrophages, preventing them from radical-induced membrane hyperpolarization, mitochondria malfunction, and phagocytosis suppression, even boosting their immune function [31,46].

The antiviral activity of the BLG polysaccharide nanoparticle may be derived from either carrying bioactive compounds or their unique physicochemical properties. It has been revealed that some food-derived nanoparticles display biological functions by carrying bioactive compounds, for instance, NPs load the majority of ephedrine (99.7%) and pseudoephedrine (95.5%) in the Ma-Xin-Shi-Gan decoction [6], the nanoparticle from freshwater clam soup carried multiple hepatoprotective bioactive, e.g., taurine, ornithine, and phytosterols [46]. In this study, epigoitrin, which was considered as the main antiviral component and a marker of antiviral efficacy of Ban-Lan-Gen in the Chinese Pharmacopeia 2010 edition [44], was screened through all the fractions. The results indicated that the polysaccharide nanoparticles with antiviral activity do not carry epigoitrin. However, whether other antiviral compounds, including certain *Radix Isatidis* polysaccharides, which have antiviral activity [47], are encapsulated into these polysaccharide nanoparticles remains unknown yet, and further study is needed for clarification.

On the other hand, the small size of nanoparticles endows them with unique physicochemical properties and consequent physiological effects [8]. The engineered nanoparticles, such as quantum dots, gold and silver nanoparticles, nanoclusters, carbon dots, graphene oxide, silicon materials, polymers, and dendrimers, can possess antiviral ability without carrying any antiviral agent having certain nanoparticle properties such as size, shape, and zeta surface potential [48]. Besides, the biological activities of some food-derived nanoparticles, such as nanoparticles from porcine bone soup, appear to rely on their unique physicochemical properties rather than carrying bioactive.

However, the obtained six nanoparticle fractions have high similarities in most of the physicochemical properties, e.g., compositions, surface charge, and stimuli responses, but were distinct in mean diameter sizes. The results of the MTT assay showed a clear correlation between the particle size of BLG nanoparticles and their cytotoxicity towards MDCK cells. Smaller nanoparticles exhibited higher cytotoxicity within the size range of 56 to 300 nm. Compared with engineered nanoparticles, which usually exhibit TC_50_ concentrations at micrograms per milliliter [49,50], BLG nanoparticles showed much milder cytotoxicity towards MDCK cells. Regarding the results of both cytotoxicity and antiviral activity, it appeared that the bioactivities of BLG nanoparticles are correlated with the physicochemical properties, especially the particle size.

Considering the complexity in constituent components and diversity in morphology, it is extraordinarily difficult to establish a relationship between the physicochemical properties of these food-derived nanoparticles and their biological activities. To address this issue, we attempted to isolate nanoparticles with different physicochemical properties, e.g., particle size [6] and surface charge [32] from soups by the means of chromatography, and evaluate their biological activities separately. As a small piece of the whole puzzle, the conclusion on the relevance between the particle size of BLG nanoparticles and their antiviral activity has certain limitations, which needs to be further confirmed by simulants of BLG polysaccharide nanoparticles.

## 5. Conclusions

In conclusion, six groups of polysaccharide nanoparticles were successfully isolated from a BLG decoction according to their particle size by the means of size-exclusive chromatography. All of the obtained nanoparticles have a high content of polysaccharides, which distinguishes them from the disclosed BLG protein nanoparticles. They also have high similarities in other compositions, surface charge, and stimuli responses. However, these nanoparticles with sizes ranging from 57 to 300 nm exhibited distinct cytotoxicity towards MDCK cells and antiviral activities against influenza virus H1N1 in vitro. It suggested that the antiviral efficacy of BLG decoction can be attributed to its nanoparticles, besides its well-known phytochemicals. It also implied that the biological effects of these polysaccharide nanoparticles, including cytotoxicity and antiviral activity, are correlated with the physicochemical properties, particularly the particle size.

## Figures and Tables

**Table 1:** Characteristics of isolated colloidal particle fractions.

Fraction	F1	F2	F3	F4	F5	F6
Ave. Size (d.nm)	300 ± 5.4	162 ± 3.8	122 ± 2.9	98 ± 2.5	67 ± 2.3	56 ± 2.2
Zeta potential (mV)	−8.2 ± 0.3	−9.2 ± 0.4	−9.5 ± 0.4	−9.8 ± 0.4	−8.5 ± 0.3	−8.6 ± 0.3
Dry matter (mg)	50.1 ± 2.2	43.7 ± 1.4	43.2 ± 1.6	46.6 ± 1.3	32.5 ± 0.7	17.3 ± 0.4
Total polysaccharides (mg)	49.9 ± 1.5	42.9 ± 1.1	42.6 ± 0.9	45.6 ± 0.8	32.2 ± 0.6	16.9 ± 0.3
Total amino acids (mg)	0.21 ± 0.07	0.17 ± 0.05	0.17 ± 0.04	0.12 ± 0.03	0.27 ± 0.06	ND

ND is for no detection.

**Table 2:** Calculated TC_50_ concentrations of BLG nanoparticle fraction.

Fraction	F1	F2	F3	F4	F5	F6
TC_50_ (mg/mL)	2.69 ± 0.23	1.81 ± 0.18	2.32 ± 0.15	2.41 ± 0.18	1.88 ± 0.38	0.67 ± 0.06

## Data Availability

The data presented in this study are available on request from the corresponding author.
